# The economic burden of asthma and chronic obstructive pulmonary disease and the impact of poor inhalation technique with commonly prescribed dry powder inhalers in three European countries

**DOI:** 10.1186/s12913-016-1482-7

**Published:** 2016-07-12

**Authors:** A. Lewis, S. Torvinen, P. N. R. Dekhuijzen, H. Chrystyn, A. T. Watson, M. Blackney, A. Plich

**Affiliations:** Covance Market Access, London, UK; Teva Pharmaceuticals Europe B.V, Haarlem, Netherlands; Department of Pulmonary Diseases, Radboud University Nijmegen Medical Centre, Nijmegen, Netherlands; Talmedica Ltd., Rossendale, and Faculty of Human and Health Sciences, University of Plymouth, Plymouth, United Kingdom

**Keywords:** Asthma, Burden of illness, COPD, Cost, DPI, Model, Poor inhalation technique

## Abstract

**Background:**

Asthma and chronic obstructive pulmonary disease (COPD) are common chronic inflammatory respiratory diseases, which impose a substantial burden on healthcare systems and society. Fixed-dose combinations (FDCs) of inhaled corticosteroids (ICS) and long-acting β_2_ agonists (LABA), often administered using dry powder inhalers (DPIs), are frequently prescribed to control persistent asthma and COPD. Use of DPIs has been associated with poor inhalation technique, which can lead to increased healthcare resource use and costs.

**Methods:**

A model was developed to estimate the healthcare resource use and costs associated with asthma and COPD management in people using commonly prescribed DPIs (budesonide + formoterol Turbuhaler^®^ or fluticasone + salmeterol Accuhaler^®^) over 1 year in Spain, Sweden and the United Kingdom (UK). The model considered direct costs (inhaler acquisition costs and scheduled and unscheduled healthcare costs), indirect costs (productive days lost), and estimated the contribution of poor inhalation technique to the burden of illness.

**Results:**

The direct cost burden of managing asthma and COPD for people using budesonide + formoterol Turbuhaler^®^ or fluticasone + salmeterol Accuhaler^®^ in 2015 was estimated at €813 million, €560 million, and €774 million for Spain, Sweden and the UK, respectively. Poor inhalation technique comprised 2.2–7.7 % of direct costs, totalling €105 million across the three countries. When lost productivity costs were included, total expenditure increased to €1.4 billion, €1.7 billion and €3.3 billion in Spain, Sweden and the UK, respectively, with €782 million attributable to poor inhalation technique across the three countries. Sensitivity analyses showed that the model results were most sensitive to changes in the proportion of patients prescribed ICS and LABA FDCs, and least sensitive to differences in the number of antimicrobials and oral corticosteroids prescribed.

**Conclusions:**

The cost of managing asthma and COPD using commonly prescribed DPIs is considerable. A substantial, and avoidable, contributor to this burden is poor inhalation technique. Measures that can improve inhalation technique with current DPIs, such as easier-to-use inhalers or better patient training, could offer benefits to patients and healthcare providers through improving disease outcomes and lowering costs.

**Electronic supplementary material:**

The online version of this article (doi:10.1186/s12913-016-1482-7) contains supplementary material, which is available to authorized users.

## Background

### The burden of asthma and chronic obstructive pulmonary disease (COPD) in Europe

Asthma and COPD are common chronic inflammatory respiratory diseases affecting 45 and 23 million people across Europe in 2011, respectively [1]. Respiratory diseases are the third leading cause of death in the European Union (EU) and have a considerable negative impact on patients’ physical and psychological wellbeing [2–5], imposing a substantial burden on healthcare providers and society as a whole [6].

Asthma and COPD comprise approximately 78 % of total direct healthcare costs associated with managing respiratory diseases in the EU, amounting to €42.8 billion in 2011 [7]. The economic burden of asthma and COPD increases markedly when indirect costs – such as those associated with lost productivity and carer time – are considered. In Europe, the annual indirect costs of asthma and COPD are approximately equal to the direct healthcare costs, totalling €14.4 billion and €25.1 billion in 2011, respectively [7].

### Treatment of asthma and COPD

There are a broad range of options available for the management of asthma and COPD. Controller medicines, such as inhaled corticosteroids (ICS), long-acting muscarinic antagonists (LAMA), long-acting β_2_ agonists (LABA) and anti-immunoglobulin E (anti-IgE), are taken preventatively to manage asthma and COPD – although the effectiveness of anti-IgE has been questioned [8]. In contrast, short-acting muscarinic antagonists (SAMA) and short-acting β_2_ agonists (SABA) are used as rescue medications to provide immediate relief from exacerbations [9, 10]. For patients with persistent asthma and COPD, global clinical guidelines recommend treatment with a fixed-dose combination (FDC) of ICS + LABA, either as a controller medication with as-needed SABA as rescue medication, or as both controller and rescue medication [9, 10].

Asthma and COPD medicines are commonly administered using either a pressurised metered dose inhaler (pMDI) or a dry powder inhaler (DPI) [9]. pMDIs function by user activation of a pressurised propellant [11], requiring a degree of dexterity, skill and training to co-ordinate actuation and inhalation in order to deliver the correct dose [9, 12]. DPIs are breath-actuated [11], with little hand-breath co-ordination required, making them easier to use than pMDIs [9, 13, 14], and are typically recommended over pMDIs [15, 16]. Clinicians and guidelines from international bodies recognise that the choice of medicine and inhaler is critical for achieving successful management of asthma and COPD [15–17].

### Poor inhalation technique

Critical inhaler errors – defined as errors which significantly reduce, or prevent entirely, deposition of medicine in the lungs [18] – can be considered a measure of poor inhalation technique. In 2011, Melani and colleagues published results of a three-month, cross-sectional study of 1,664 Italian asthma and COPD patients using DPIs, which found that 44 % of people using budesonide + formoterol (BF) Turbuhaler^®^ (Symbicort^®^ Turbuhaler^®^) and 34 % of people using fluticasone + salmeterol (FS) Accuhaler^®^ (Seretide^®^ Accuhaler^®^) had poor inhalation technique [19]. Moreover, a systematic review of patients with asthma and COPD found that up to 94 % of DPI users made at least one inhaler error when examined by a healthcare professional (HCP) [20].

Importantly, HCPs may also demonstrate poor inhalation technique. Independent studies from multiple countries have shown that at least a third of – and in some cases all – HCPs performed at least one critical error with pMDIs and DPIs [21–25]. Similarly, a review of 20 studies of pMDI and DPI use found that more than three quarters of nurses, and over a third of respiratory specialists, did not perform all stages of inhalation correctly [26]. The frequency with which HCPs can demonstrate poor inhalation technique indicates that commonly prescribed inhalers are difficult to use. As HCPs are charged with teaching patients to use inhalers effectively, poor inhalation technique among HCPs may result in patients receiving incorrect or inconsistent advice and training.

Studies from many countries have shown that poor inhalation technique correlates with reduced disease control and increased use of healthcare resources [16, 27], which in turn negatively impacts patient health-related quality of life (HRQoL) [28]. Therefore, poor inhalation technique presents a potentially considerable, and avoidable, burden to healthcare organisations and patients alike. Although it is widely accepted that inhalation technique is a significant factor in the control of respiratory disease [29], its contribution to the cost of asthma and COPD management has not been quantified. An economic model was designed to assess the healthcare and societal burden of managing asthma and COPD using DPIs containing ICS + LABA FDCs, and how this may be impacted by poor inhalation technique.

## Methods

### Model design

A burden-of-illness model was developed from a societal perspective for Spain, Sweden and the United Kingdom (UK). These countries were chosen in order to give a range of population sizes, geographical locations and economies. The model assessed the economic burden of managing asthma and COPD using either BF Turbuhaler^®^ or FS Accuhaler^®^ over 1 year, and estimated the contribution of poor inhalation technique to this burden. These inhalers were chosen as they are the most commonly prescribed DPIs in Europe [30].

Direct and indirect costs were included in the model. Direct costs included inhaler acquisition costs, scheduled healthcare costs (visits to nurses, general practitioners (GPs), and specialists) and unscheduled healthcare costs (hospitalisations, emergency department (ED) visits, and additional courses of antimicrobials or oral corticosteroids (OCS)). Indirect costs were determined using the number of productive days lost due to asthma or COPD. Costs for Sweden and the UK were converted to Euro using historical exchange rates [31]. All costs were inflated to 2015 values based on healthcare-specific consumer price indices (CPIs) [32–34], except the cost of each lost productive day, which was inflated using national CPIs [35].

### Parameters

#### Model population

Adult asthma or COPD patients using BF Turbuhaler^®^ or FS Accuhaler^®^ were included in the analysis. This population was estimated based on the number of individuals aged 18 years and older with diagnosed asthma or COPD, according to population estimates from national statistical databases [36–38] and epidemiological data from national regulatory bodies [39–42]. The proportion of patients receiving FDCs of ICS + LABA to manage their asthma or COPD was calculated based on data from national regulatory bodies and published studies [39–41, 43, 44]. The annual number of patients receiving BF Turbuhaler^®^ or FS Accuhaler^®^, at each delivered dose strength, was estimated using 2014 national sales data (moving annual total; a rolling measure of data from the past year taken every month) [30] (Table 1).

**Table 1:** Model populationPlease check if "Tables 1-6 data" were presented correctly.The data in these tables are correct. We have changed the formatting of the tables to make them easier to readPlease left align text in the left column of table 6.

Parameter	Spain	Sweden	UK
Prevalence			
Total number of individuals aged ≥18 (*n*)	37,860,506 [36]	7,772,932 [37]	50,909,098 [38]
Prevalence of diagnosed asthma (%)	3.5 [39]	8.0 [41]	6.1 [42]
Prevalence of diagnosed COPD (%)	2.8 [40]	7.0 [43]	1.8 [42]
Proportion of patients receiving ICS + LABA FDCs (%)			
Asthma	33.4 [39]	50.0 [41]	35.5 [44]
COPD	33.7 [40]	39.7 [43]	35.5^a^
Proportion of patients using commonly prescribed DPIs to administer ICS + LABA FDCs (%)			
BF Turbuhaler^®^	34.6 [30]	74.7 [59]	31.1 [30]
FS Accuhaler^®^	37.3 [30]	15.1 [59]	25.2 [30]
Prescription distribution of BF Turbuhaler^®^ doses (%)			
BF Turbuhaler^®^ 80/4.5 μg	3.4 [30]	1.1 [30]	8.6 [30]
BF Turbuhaler^®^ 160/4.5 μg	54.0 [30]	48.5 [30]	55.9 [30]
BF Turbuhaler^®^ 320/9 μg	42.7 [30]	50.4 [30]	35.5 [30]
Prescription distribution of FS Accuhaler^®^ doses (%)			
FS Accuhaler^®^ 100 μg	5.9 [30]	3.8 [30]	11.5 [30]
FS Accuhaler^®^ 250 μg	40.1 [30]	50.3 [30]	25.5 [30]
FS Accuhaler^®^ 500 μg	54.0 [30]	45.9 [30]	63.0 [30]

BF Turbuhaler^®^ is marketed as Symbicort^®^ Turbohaler^®^ in the UK, and Symbicort^® ^Turbuhaler^®^ in Spain and Sweden; FS Accuhaler^®^ is marketed as Seretide^®^ Accuhaler^®^ in Spain and the UK, and Seretide^®^ Diskus^®^ in Sweden. Values are subject to rounding

^a^Assumed to be equal to proportion of asthma patients

#### Inhaler acquisition costs

Costs of BF Turbuhaler^®^ and FS Accuhaler^®^ in Spain, Sweden and the UK were sourced from the Ministry of Health [45], national sales data (moving annual total) [30] and the Monthly Index of Medical Specialities (MIMS) [46, 47], respectively (Table 2).

**Table 2:** Cost per device

Parameter	Spain (€)	Sweden (€)	UK (€)
BF Turbuhaler^®^			
BF Turbuhaler^®^ 80/4.5 μg	32.92 [45]	53.30 [30]	45.60 [46]
BF Turbuhaler^®^ 160/4.5 μg	41.46 [45]	42.53 [30]	52.65 [46]
BF Turbuhaler^®^ 320/9 μg	41.46 [45]	38.61 [30]	52.65 [46]
FS Accuhaler^®^			
FS Accuhaler^®^ 100 μg	29.38 [45]	25.84 [30]	24.88 [47]
FS Accuhaler^®^ 250 μg	35.50 [45]	30.48 [30]	48.51 [47]
FS Accuhaler^®^ 500 μg	47.90 [45]	40.12 [30]	56.38 [47]

Exchange rates used were GBP/EUR 0.74 and SEK/EUR 9.40

#### Resource use and healthcare costs

Direct and indirect healthcare events and costs are displayed in Table 3. The majority of resource use inputs and all costs were derived from country-specific sources, such as national or regional registries and peer-reviewed articles; where necessary, reasonable assumptions were made to utilise applicable data.

**Table 3:** Direct and indirect events and costs

Parameter	Spain		Sweden		UK	
	Frequency (*n*)	Cost per event (€)^a^	Frequency (*n*)	Cost per event (€)^a^	Frequency (*n*)	Cost per event (€)^a^
Annual scheduled healthcare events per person						
Asthma						
Nurse visits	0.76 [60]	18.99 [61]	0.68 [62]	62.08 [63]	0.85 [64–66]	31.35 [67]
GP visits	2.30 [68]	39.35 [61]	0.68 [62]	152.41 [63]	0.60 [64–66]	75.51 [67]
Specialist visits	2.21 [68]	78.70 [60]	0.34 [62]	206.25 [63]	0.15 [69]^b^	133.93 [67]
COPD						
Nurse visits	0.76 [60]	18.99 [61]	0.00^c^	0.00^c^	1.05 [64, 66]	31.35 [67]
GP visits	0.47 [68]	39.35 [61]	1.70 [70]	152.41 [63]	1.30 [64, 66]	75.51 [67]
Specialist visits	1.43 [68]	78.70 [60]	1.70 [70]	206.25 [63]	3.42 [71]	133.93 [67]
Annual unscheduled healthcare events per person						
Asthma						
Hospitalisations	0.09 [60, 68]	4,495.90 [61, 68]	0.12 [62, 72]^d^	748.15 [63, 72]^d^	0.02 [72, 73]	1,753.68 [74]
ED visits	0.26 [60, 68]	181.62 [61, 68]	0.20 [75]	177.67 [63]	0.02 [72, 73]	182.12 [74]
Antimicrobial courses	0.70 [76]^e^	4.76 [77]	0.50^f^	1.07 [78]	0.70 [76]^e^	25.65 [79, 80]
OCS courses	0.63 [81]	17.22 [77]	0.20^f^	2.34 [78]	0.14 [81]	55.28 [82]
COPD						
Hospitalisations	0.26 [60, 68]	3,448.13 [49, 61]	0.38 [70, 72]^d^	1,915.26 [63, 72]^d^	0.12 [72, 73]	3,554.73 [74]
ED visits	0.08 [60, 68]	181.62 [49, 61]	0.31 [43]	177.67 [63]	0.12 [72, 73]	182.12 [74]
Antimicrobial courses	0.38 [83]	4.76 [77]	2.00^f^	1.07 [78]	1.51 [83]	2.94 [84, 85]
OCS courses	0.17 [83]	17.22 [77]	1.60^f^	2.34 [78]	0.68 [83]	55.28 [82]
Annual productivity losses per person						
Productive days lost (asthma)	12.00 [86, 87]	62.04 [36]	4.00 [62]	205.50 [88]	17.00 [89, 90]	169.22 [91]
Productive days lost (COPD)	24.00^g^	62.04 [36]	24.00^g^	205.50 [88]	24.00 [73, 92]	169.22 [91]

^a^All cost values are inflated to May 2015 figures, and converted to Euro, where appropriate. ^b^Data reported by an American cohort study of members of a managed care organisation [69] – assumed to be representative of the UK. ^c^Patients with severe COPD in Sweden receive outpatient care from GPs and specialists, and do not visit nurses (based on an interview with a clinical expert).^d^Calculated using average length of stay data from UK hospitals [70]. ^e^Data reported by a study of Irish GP practices [76] – assumed to be representative of Spain and the UK. ^f^ Values based on the opinion of a clinical expert. ^g^Data assumed to be the same as reported for the UK. Values are subject to rounding

#### Impact of poor inhalation technique

The proportion of patients demonstrating poor inhalation technique with BF Turbuhaler^®^ (43.5 %) and FS Accuhaler^®^ (34.5 %) was based on the study by Melani and colleagues [19]. It was assumed that the increased risk of unscheduled healthcare events over baseline due to poor inhalation technique reported in this Italian study [19] was applicable to other European countries (Table 4). We conservatively assumed that the increased risk of lost productivity due to poor inhalation technique was equal to the lowest risk increase reported for any other event (ie hospitalisation).

**Table 4:** Increased risk of unscheduled healthcare events associated with poor inhalation technique

Unscheduled healthcare event	Increased risk^a^
Hospitalisation	47 %
ED visit	62 %
Course of antimicrobials	50 %
Course of OCS	54 %
Productive day lost	47%^b^

^a^Based on the increased risk over patients with correct inhaler technique (odds ratio) of at least one critical inhaler error and self-reported utilisation of healthcare resources used in the year since the critical inhaler error [19]

^b^Conservatively assumed to be equal to the lowest increased risk reported for any unscheduled healthcare event (hospitalisation)

#### Sensitivity analyses

One-way sensitivity analyses were performed using upper and lower bounds based on reported values where possible – if no data were available bounds were set at ±20 %. The following parameters were varied:

- Proportion of patients using ICS + LABA FDCs (±10 %) – variation accounts for changes in prescription habits
- Number of doses per day – the upper (3) and lower (1) bounds reflect recommendations in the Summary of Product Characteristics (SmPCs) for each inhaler
- Cost of hospitalisation (±20 %)
- Cost of ED visits (±20 %)
- Cost of additional courses of antimicrobials (±20 %)
- Cost of additional courses of OCS (±20 %)
- Proportion of patients with poor inhalation technique (±20 %)

## Results

### Number of events

The annual number of events is shown in Table 5. The largest eligible patient population was in the UK, followed by Spain and Sweden. The number of productive days lost due to asthma and COPD aligned with the relative size of the eligible population for each country, and comprised 83.8–93.8 % of the total number of all reported events in each country.

**Table 5:** Total annual number of events due to asthma and COPD

Output	Spain	Sweden	UK
Population (adults receiving BF Turbuhaler^®^ or FS Accuhaler^®^)	572,317	473,022	803,821
Number of scheduled healthcare events			
Nurse visit	434,961	189,795	719,878
GP visit	854,662	519,445	610,497
Specialist visit	1,068,044	424,548	721,953
Total	2,357,668	1,133,788	2,052,328
Number of unscheduled healthcare events			
Hospitalisation	92,676	106,546	33,851
ED visits	101,035	115,077	33,715
Antimicrobial courses	313,760	524,096	699,262
OCS courses	239,520	363,655	207,711
Total	746,991	1,109,374	974,539
Number of productive days lost			
Lost productivity	9,895,128	5,736,196	14,712,035

Values are subject to rounding

### Costs

**Fig. 1:**
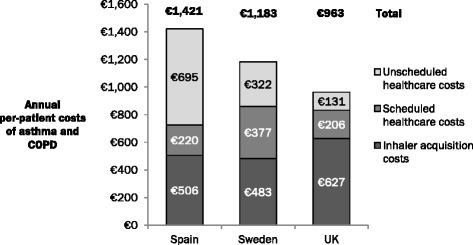
Annual direct per-patient costs of asthma and COPD. The annual per-patient costs of asthma and COPD were calculated by dividing the total annual costs by the number of eligible patients in the model. Values are subject to rounding

The estimated total direct costs of asthma and COPD in 2015 were predicted to be €813 million, €560 million and €774 million in Spain, Sweden and the UK, respectively. Despite having the largest eligible population, the costs of asthma and COPD per patient were lowest in the UK, while the highest per-patient costs were incurred in Spain (Fig. 1). Inclusion of indirect costs increased the burden of asthma and COPD substantially, with the costs per-patient rising to €2,474, €3,675 and €4,060 in Spain, Sweden and UK, respectively, resulting in total annual costs of €1.4 billion, €1.7 billion and €3.3 billion, respectively. Despite patients in Spain losing more productive days on average compared with patients in Sweden, total annual indirect costs in Spain were approximately half of those for Sweden (€0.60 billion compared with €1.18 billion, respectively).

### Poor inhalation technique

The contribution of poor inhalation technique to the burden of asthma and COPD is summarised in Table 6. Across the three countries studied, 15.4–20.7 % of unscheduled healthcare events and costs were attributable to poor inhalation technique.

**Table 6:** Costs of poor inhalation technique for patients using BF Turbuhaler^®^ and FS Accuhaler^®^

Unscheduled healthcare events	Spain		Sweden		UK	
	Frequency (n; thousands)	Cost (€; millions)	Frequency (n; thousands)	Cost (€; millions)	Frequency (n; thousands)	Cost (€; millions)
Hospitalisations						
Total	92.7	373.9	106.5	130.7	33.9	73.3
Not due to poor inhalation technique	78.4	316.2	89.0	109.2	28.6	61.8
Due to poor inhalation technique^a^	14.3	57.7	17.6	21.5	5.3	11.5
Contribution of poor inhalation technique^b^(%)	15.4		16.5		15.6	
ED visits						
Total	101.0	18.4	115.1	20.4	33.7	6.1
Not due to poor inhalation technique	81.4	14.8	91.3	16.2	27.1	4.9
Due to poor inhalation technique^a^	19.6	3.6	23.8	4.2	6.6	1.2
Contribution of poor inhalation technique^b^(%)	19.4		20.7		19.7	
Antimicrobial courses						
Total	313.8	1.5	524.1	0.6	699.3	14.3
Not due to poor inhalation technique	262.7	1.3	433.2	0.5	584.0	12.0
Due to poor inhalation technique^a^	51.0	0.2	90.9	0.1	115.3	2.4
Contribution of poor inhalation technique^b^(%)	16.3		17.3		16.5	
OCS courses						
Total	239.5	4.1	363.7	0.9	207.7	11.5
Not due to poor inhalation technique	198.0	3.4	296.4	0.7	171.2	9.5
Due to poor inhalation technique^a^	41.5	0.7	67.2	0.2	36.5	2.0
Contribution of poor inhalation technique^b^(%)	17.3		18.5		17.6	
Productive days lost						
Total	9,714.9	602.7	5,736.2	1,178.8	14,712.0	2,489.6
Not due to poor inhalation technique	8,215.5	509.7	4,790.8	984.5	12,409.8	2,100.0
Due to poor inhalation technique^a^	1,499.4	93.0	945.4	194.3	2,302.2	389.6
Contribution of poor inhalation technique^b^(%)	15.4		16.5		15.6	
Overall cost burden						
Total	10,461.9	1,000.6	6,845.6	1,331.4	15,686.6	2,594.8
Not due to poor inhalation technique	8,836.0	845.4	5,700.7	1,111.1	13,220.7	2,188.2
Due to poor inhalation technique^a^	1,625.8	155.2	1,144.9	220.3	2,465.9	406.7

^a^The number of unscheduled healthcare events associated with poor inhalation technique is based on the increased risk of each event as reported by Melani and colleagues [19], taking account of the proportion of patients experiencing an event who have: i) good inhalation technique; ii) poor inhalation technique, but the reason for the event is not due to poor inhalation technique; iii) poor inhalation technique, and the poor inhalation technique is the cause of the event. The cost of poor inhalation technique was calculated by multiplying the number of events occurring per patient due to poor inhalation technique by the weighted cost of the event.^b^Total number of unscheduled healthcare events and costs. Values are subject to rounding

**Fig. 2:**
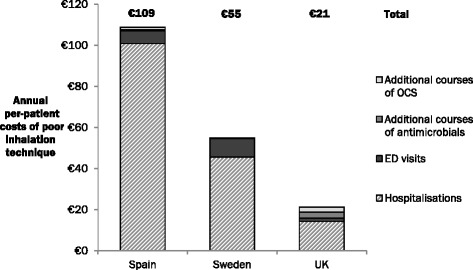
Annual direct per-patient costs of poor inhalation technique. Annual per-patient costs of poor inhalation technique were calculated by dividing the total annual costs of events attributable to poor inhalation technique by the number of patients included in the model. Values are subject to rounding

Figure 2 reveals that the per-patient costs of unscheduled healthcare events due to poor inhalation technique were highest in Spain (€109), followed by Sweden (€55) and the UK (€21). Per-patient costs in Spain were substantially higher than in the other two countries due to the high costs of hospitalisation. The contribution of additional courses of antimicrobials and OCS to the cost burden of poor inhalation technique in Spain and Sweden was negligible; however, in the UK, these costs were each greater than the costs of ED visits.

The total cost burden of poor inhalation technique more than doubled when productivity losses were taken into account (Table 6). These indirect costs were highest in the UK (€390 million), followed by Sweden (€194 million) and Spain (€93 million). Inclusion of indirect costs increased the total per-patient costs of poor inhalation technique to €271 in Spain, €466 in Sweden and €506 in the UK.

### Sensitivity analyses

**Fig. 3:**
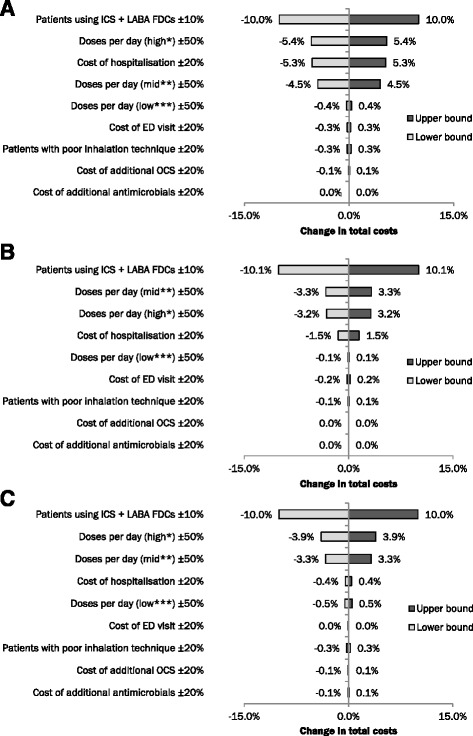
One-way sensitivity analyses. *Patients prescribed 320/9 μg or 500 μg inhalers, **Patients prescribed 160/4.5 μg or 250 μg inhalers, ***Patients prescribed 80/4.5 μg or 100 μg inhalers. Sensitivity analyses for **a**) Spain, **b**) Sweden and **c**) the UK. Parameters were varied as described in the Methods section. Results are displayed from the greatest change to the least change for each country. Values are subject to rounding

The results of the one-way sensitivity analyses are shown in Fig. 3. The model was most sensitive to the proportion of patients using ICS + LABA, and moderately sensitive to the number of doses per day and the cost per hospitalisation. The ranking of each parameter was similar across the three countries; with regards to Spain, the model was more sensitive to the costs of hospitalisation than to the number of doses per day for mid-strength inhalers, while the model in Sweden was more sensitive to the number of doses per day for mid strength inhalers than high strength inhalers.

## Discussion

We developed a model to estimate the burden of managing asthma and COPD with the most commonly prescribed DPIs in Spain, Sweden and the UK. Our analysis estimated the burden to be substantial, with 572,317, 473,022 and 803,821 adults using BF Turbuhaler^®^ or FS Accuhaler^®^ in Spain, Sweden and the UK, respectively. Given the population size differences between countries, the proportion of adults included in the model for Sweden was high relative to those values estimated for Spain and the UK – this was likely due to a higher prevalence of asthma and COPD, and higher prescription rates of BF Turbuhaler^®^.

A total of 5.5 million scheduled and 2.8 million unscheduled healthcare events were estimated to occur across the three countries annually. The highest number of scheduled healthcare events was estimated to occur in Spain, primarily driven by a higher incidence of specialist visits compared with other countries. The highest number of unscheduled healthcare events occurred in Sweden, despite it having the lowest eligible patient population among the countries studied; this may be due to the high prescription rates of BF Turbuhaler^®^, as patients using this inhaler have a higher risk of incurring unscheduled healthcare events than patients using FS Accuhaler^®^ [19].

The model estimated direct per-patient costs (inhaler acquisition costs, scheduled healthcare costs and unscheduled healthcare costs) of disease management in Spain, Sweden and the UK to be €1,421, €1,183 and €963, respectively. These values are in broad agreement with previously published cost estimates. For example, in 2007, a prospective observational study of 627 asthma patients in Spain reported a direct annual cost of €1,533 per patient [48], while in 2003 a separate, multicentre, epidemiological study of 10,711 Spanish COPD patients determined the cost per patient to be €1,922 [49]. When inflation is applied to these values, the reported annual per-patient costs of asthma and COPD in Spain are €1,675 and €2,111, respectively [32]. Minor differences in cost estimates compared with our results are likely due to variations in methods of data collection and analysis, treatment regimens, patient populations and disease severity, and regional healthcare costs.

In the UK, the direct cost burden of asthma and COPD was reported to be £1.8 billion in 2012 [50], which, when converted to Euro and inflated to 2015 values [31], equates to €2.6 billion. Our analysis estimated the direct cost of managing patients using BF Turbuhaler^®^ or FS Accuhaler^®^ – who represent 20 % of the total UK asthma and COPD patient population – to be €774 million, which is approximately 29.8 % of the reported total costs for the UK. Therefore, assuming the two studies are comparable, patients considered by our model have higher than average costs of asthma and COPD management, which is to be expected as these patients have persistent forms of disease.

The model showed that the indirect costs of asthma and COPD exceeded the direct costs in Sweden and the UK, while the two were approximately equal in Spain. This is similar to results reported by the European Respiratory Society (ERS), which stated that direct and indirect costs for the whole of Europe were approximately equal [7]. Estimating the indirect costs associated with a disease is challenging, as they are not well defined and can be calculated in multiple ways. Whilst disability and family carer costs add to the indirect burden of disease [7], these costs were not included in our analysis. Instead, productivity losses were calculated based solely on the number of work days lost and the average salary within each country. Our estimation of the indirect burden of asthma and COPD is therefore conservative, and the true costs would likely eclipse those reported here.

Studies from many countries have shown that poor inhalation technique – which is commonly observed in users of BF Turbuhaler^®^ and FS Accuhaler^®^ [19] – correlates with reduced disease control and increased healthcare costs [16, 27]. However, to our knowledge, no in-depth attempt has previously been made to quantify the contribution of poor inhalation technique to overall healthcare costs in DPI users. Our analysis estimated the total direct costs of poor inhalation technique to be €105 million annually across the three countries, amounting to 4.9 % of the total direct costs of asthma and COPD management. When lost productivity was included, the total costs of poor inhalation technique rose to €782 million (12.2 %) in Spain, Sweden and the UK. Poor inhalation technique therefore represents a substantial budgetary and societal burden.

Several studies have shown that patient inhalation technique can be improved with additional training [27, 51, 52], however, regular check-ups are required to maintain correct inhalation technique over time [51]. Additionally, HCPs charged with teaching patients to use inhalers correctly often demonstrate poor inhalation technique themselves [26]. Education of HCPs and patients is an important step in improving the management of asthma and COPD [53]; however, training alone is unlikely to be sufficient for achieving optimal control [54]. Consequently, the introduction of novel, easy-to-use inhalers that have the potential to improve patient inhalation technique will play an important role in optimising control of respiratory diseases [54]. Inhalers that are more intuitive to use or require less dexterity than BF Turbuhaler^®^ or FS Accuhaler^®^ could reduce the likelihood of patients making critical inhaler errors [54], which would lower the risk of unscheduled healthcare events [19], and – provided acquisition costs of these novel inhalers are comparable to currently prescribed DPIs – therefore result in direct cost savings. Indeed, correct choice of inhaler is seen as a critical factor in the management of asthma and COPD [15–17], as increased patient satisfaction with an inhaler is associated with improved adherence to treatment and enhanced disease control [55]. Introduction of novel inhalers that address current unmet needs could therefore reduce the patient and economic burden associated with asthma and COPD management.

Sensitivity analyses showed that the model was most sensitive to the proportion of patients using ICS + LABA, and moderately sensitive to the number of doses per day and the costs per hospitalisation; variations among the remaining inputs had little effect on the output of the model. We therefore conclude that the model is robust. However, there are a number of limitations to the model. Firstly, the model does not consider the frequency with which patients take their medication, the impact of patient adherence or comorbidities. Whilst poor patient adherence is associated with increased use of healthcare resources [56], the absence of data supporting the contribution of adherence to the different costs considered in this analysis meant that we did not factor adherence into the model calculations. Comorbidities contribute to the number of exacerbations [57] and, subsequently, days of lost productivity [58] experienced by patients; however, the costs of comorbidities are hard to quantify, and were therefore not included in this analysis. This, together with the exclusion of patient adherence, likely results in conservative estimates of asthma and COPD management costs.

Secondly, visits to nurses, GPs and specialists were based on the total number of visits per year. The model considered all of these visits to be scheduled healthcare events, though in actuality some of these visits would be due to exacerbations and would therefore be classed as unscheduled healthcare events. As such, the model may overestimate the cost of scheduled healthcare events and underestimate the cost of unscheduled healthcare events and, consequently, our reported burden of poor inhalation technique is likely to be conservative. Therefore, any reduction in the number of unscheduled healthcare events will likely lead to greater cost savings than would be predicted by our model.

Thirdly, the prevalence of poor inhalation technique in the countries studied was assumed to be the same as reported in Italy [19], and the increased risk of healthcare resource use due to poor inhalation technique was assumed to be the same across all countries. While practices perceivably vary from country to country, no studies have estimated the impact of poor inhalation technique in Spain, Sweden or the UK. This information would help provide a more accurate estimate of the costs of poor inhalation technique within these countries.

Finally, this study focusses on the impact of poor inhalation technique on unscheduled healthcare use and productivity losses only. Reduced disease control due to poor inhalation technique likely reduces patient HRQoL, which is not measured in this analysis. Melani and colleagues reported a significant association between asthma and COPD patients making at least one critical inhaler error and several patient-reported outcomes, such as limitations during everyday life, shortness of breath, use of rescue inhaler, and sleep disturbance [19]. Applying quantifiable estimates to such outcomes would emphasise further the burden of poor inhalation technique.

## Conclusions

The cost of managing asthma and COPD with DPIs is considerable. A substantial, and avoidable, contributor to this burden is poor inhalation technique with currently prescribed DPIs. Measures that can improve inhalation technique with current DPIs, such as easier-to-use inhalers and better patient training, could offer benefits to patients and healthcare providers through improved disease outcomes and lowered costs.

## Abbreviations

BF, budesonide + formoterol; COPD, chronic obstructive pulmonary disease; CPI, consumer price index; DPI, dry powder inhaler; ED, Emergency department; ERS, European Respiratory Society; EU, European Union; FDC, fixed dose combination; FS, fluticasone + salmeterol; GP, general practitioner; HCP, healthcare professional; HRQoL, health-related quality of life; ICS, inhaled corticosteroids; ISPOR, International Society for Pharmacoeconomics and Outcomes research; LABA, long-acting β_2_ agonists; LAMA, long-acting muscarinic antagonists; MIMS, monthly Index of Medical Specialities; OCS, oral corticosteroids; pMDI, pressurised metered dose inhaler; SABA, short-acting β_2_ agonists; SAMA, short-acting muscarinic antagonists; SmPC, summary of product characteristics; UK, United Kingdom

## Additional file

Additional file 1: Inputs used in the model for the analyses presented in this publication. (XLSX 20 kb)
